# Performance Activities and Match Outcomes of Professional Soccer Teams during the 2016/2017 Serie A Season

**DOI:** 10.3390/medicina55080469

**Published:** 2019-08-12

**Authors:** Umile Giuseppe Longo, Francesco Sofi, Vincenzo Candela, Monica Dinu, Matteo Cimmino, Carlo Massaroni, Emiliano Schena, Vincenzo Denaro

**Affiliations:** 1 Department of Orthopaedic and Trauma Surgery, Campus Bio-Medico University, 00128 Rome, Italy; 2 Centro Integrato di Ricerca (CIR), Campus Bio-Medico University, 00128 Rome, Italy; 3 Department of Experimental and Clinical Medicine, University of Florence, 50121 Florence, Italy; 4 Don Carlo Gnocchi Foundation, Onlus IRCCS, 20148 Florence, Italy; 5 Unit of Measurements and Biomedical Instrumentation, Department of Engineering, Università Campus Bio-Medico di Roma, Via Alvaro del Portillo, 21, 00128 Rome, Italy

**Keywords:** soccer, elite, performance, anthropometry, sport, championship

## Abstract

*Background and Objectives:* Soccer is the most popular sport in the world. To describe athletic performance, match statistics, and their relationships with the probability of achieving the first positions of the final ranking in the Italian football league “Serie A”, season 2016/2017. *Materials and Methods:* Analyses comprised all the matches played by the 20 teams of the “Serie A” championship during the season 2016–2017. Indicators of athletic performance (total distance covered in km, jogging, running and sprint activities, and average speed) and match statistics (total shots, shots on target, goal attempts, assists, turnovers, and steals) were obtained from the Italian football league. *Results:* Analyses of performance activities according to the final ranking showed no significant differences for the total distance covered and speed, while a statistically significant difference (*p* < 0.05) among teams was observed for jogging, running and sprint activities. In regard to match statistics, all the parameters investigated were significantly different among the teams. By grouping teams into four subgroups (those who qualified for the Champions League, those who qualified for the Europe League, those who ranked intermediate positions and those who relegated from the “Serie A” league), the percentage of jogging, running and sprint activities, as well as match statistics were significantly different among groups, with a downward trend for total shots, shots on target, goal attempts, assists, and turnovers. The logistic regression analysis revealed that sprint activities as well as total shots, shots on target, goal attempts, and assists higher than the 3rd tertile of their distribution were associated with a higher probability of reaching the first three positions of the final ranking. *Conclusions:* An increased probability to achieve the first positions of the final ranking in the Italian football league “Serie A” seemed to be mainly related to sprint activity, goal attempts, total shots, shots on target and assists.

## 1. Introduction

Soccer is the most popular sport in the world. At a competitive level, the physiological demands of soccer implicate an increased work rate and high tactical, mental, and physiological skills [1,2,3]. Elite soccer players should have multiple physical and technical capacities [4,5]. Performance in soccer depends on a variety of individual skills and the interaction among different players within the team [6,7]. Technical responses to the physical demands of team sports are not well understood. Despite the acknowledged importance of developing physical qualities in soccer players, the analysis of player’s activities is still a relatively new concept for players and coaches. The study of player’s activities during the matches could optimise the team’s performance and contribute to the team’s success.

The aims of this study were to provide a detailed analysis of the match performance activities of the Italian soccer league “Serie A” season 2016–2017, according to the final ranking and to the match outcomes, and to analyze the total distance covered, the percentage of total distance covered at high intensity, total number of performed sprints and peak running speed attained by soccer players by quantifying the association between match running performance and success across a season in soccer teams competing within a top European league.

## 2. Materials and Methods

### 2.1. Experimental Approach to the Problem

Through a detailed analysis of professional soccer teams during the 2016/2017 “Serie A” season, the importance of athletic performance (total distance covered in km, jogging, running and sprint activities, average speed and average distance covered per minute) and match statistics (total shots, shots on target, goal attempts, assists, turnovers, and steals) in achieving the first positions of the final ranking were evaluated.

#### Match Data

“Serie A” is a professional league competition for football clubs located at the top of the Italian football league system. Each season, which runs from August to May, is played with 20 teams in a round-robin format, for a total of 38 games for each team. The present study analysed data from 380 individual matches, played during the 2016/2017 season. Technical data from each match was obtained using a video-computerized match-analysis system Digital.Stadium® VTS (SICS, Bassano, Italy) with six 25-Hz sample frequency cameras. The system consists of six fixed cameras positioned all around the pitch, calibrated and synchronized, and allow the collection of athletic performance and match statistics from all the players involved in the game. The validity and reliability of this system for taking such measurements have been described in detail elsewhere [8,9]. Recorded variables included total distance covered in km and distance covered. Based on speed ranges, three intensity zones were determined: Walking (0–2.2 m/s), running (2.2–4.4 m/s) and sprinting (>4.4 m/s).

For each match we analysed the following parameters, collected from the official match reports of the Italian Football Federation (www.legaseriea.it): Total distance covered in km, jogging, running and sprint activities, average speed, average distance covered per minute, total shots, shots on target, goal attempts, assists, turnovers, and steals. Performance activities and match outcomes were also analysed according to the result obtained at the end of the season, by dividing the population into teams that obtained the first three positions of the final ranking (i.e., positions for getting into the Champions League), teams that obtained the final ranking from the 4th to the 6th position (i.e., positions for getting into the Europe League), teams that ranked from the 7th to the 18th position, and teams that relegated from the “Serie A” championship (i.e., teams that ranked from the 18th to the 20th position). The study was conducted in compliance with the Declaration of Helsinki. Due to the nature of the study and to the type of data the study did not need approval from an ethic committee.

### 2.2. Statistical Analysis

Data were analysed using the SPSS software for Macintosh (version 20.0). For each variable, the means and their standard deviations (SD) were used as descriptive statistics. One-way ANOVA analysis was used for testing differences between means, while the Kruskal-Wallis test was used for comparisons among groups. Pearson’s correlation coefficients were calculated for the relation between physical performance and match outcomes.

To test for the probability of being in the first three and six positions of the final ranking at the end of the season, a univariate logistic regression analysis using the first three and six positions as independent variables and jogging, running, sprint, total shots, shots on target, goal attempts, assists, turnovers, and steals as covariates was performed. Results were reported as odds ratio (OR) and 95% confidence intervals (CI). Values were considered significant at *p* < 0.05.

## 3. Results

The analyses comprised all the matches performed by the teams of the “Serie A” championship during the season 2016/2017. The total distance covered by a single team per match throughout the entire season was 107.3 ± 4.5 km, of which 27.7 ± 1.7 km determined by jogging, 70.9 ± 4.7 km by running and 8.7 ± 3.2 km by sprint activities. The average speed was 6.7 ± 0.3 km/h, while the average distance covered per minute was 119.2 ± 5 m/min.

One-way ANOVA analysis of performance activities and match statistics according to the final ranking was performed in order to investigate differences among teams. As reported in Table 1, there was no significant difference among teams in the total distance covered, distance covered per minute and speed, while a statistically significant difference (*p* < 0.05) was observed for the percentage of jogging, running and sprint activities.

The average number of total shots, shots on target, goal attempts, assists, turnovers and steals, which significantly differed among teams, is shown in Table 2.

By dividing the teams according to the result obtained at the end of the season (the first three positions, from the 4th to the 6th position, from the 7th to the 18th position, and from the 18th to the 20th position), no significant differences for the total distance covered and speed were reported among the four subgroups. The percentage of jogging, running and sprint activities, as well as parameters related to the match statistics, on the contrary, were significantly different, with a downward trend for total shots, shots on target, goal attempts, assists, and turnovers (Table 3).

The correlation analyses between the physical performance and parameters related to the match statistics are reported in Table 4. While average speed was only correlated with turnovers and steals, different intensity ranges were differently correlated with shots, shots on target, goal attempts and assists. In particular, negative correlations were observed with running and positive correlations were observed with sprint activities.

Finally, in order to investigate the parameters that are most significantly associated with the probability of being in the first three and six positions of the final ranking, we conducted a logistic regression analysis by grouping the population into tertiles of distribution of the investigated parameters. While a percentage of running higher than the 3rd tertile of the whole distribution was associated with a lower probability of reaching the first three positions at the end of the season, sprint activity as well as total shots, shots on target, goal attempts, and assists was associated with a higher probability of reaching these positions (Table 5).

In particular, >9 goal attempts per match determined a seven-fold increased possibility of reaching the first three positions. On the other hand, the probability of being in the first six positions of the final ranking seemed to be less influenced by running and sprint activities, and more influenced by the match statistics, in particular total shots, shots on target, goal attempts, assists and steals.

## 4. Discussion

The aim of this study was to analyse performance activities of elite footballers of the Italian soccer league “Serie *A*” during the football match and to identify parameters that are most significantly associated with the probability of being in the first positions of the final ranking. The results of our analyses suggest that sprint activity, goal attempts, total shots, shots on target and assists were related to an increased probability to achieve the first positions of the final ranking in the Italian football league “Serie A”. A higher percentage of running activity, on the other hand, was negatively associated with shots, shots on target, goal attempts and assists, and determined a lower probability of reaching the first three positions at the end of the season.

Activity patterns in soccer matches have been investigated by different authors [10,11,12,13,14,15]. Coaches and trainers consider useful the analysis of player activity profiles to design optimal athlete training prescriptions, competition strategies, manage load and recovery and to formulate training drills to help players address the real physical challenges of the sport [7,10]. A good measurement of the players’ exercise performance is the total distance covered by a team and by individual players in match play, consisting of distances covered when walking, jogging and running at low, moderate and high intensity in different directions [3,16]. Information about the total distance covered in a match, intensity or high-intensity running, percentage of jogging, running and sprint activities may be used to represent a player’s physical load during a match [3,10].

Previous studies in elite soccer showed that players spend the majority of match time in low intensity speed ranges (standing, walking, and jogging), with high-intensity (running and sprinting) accounting for about 10% of the total time [17]. During a soccer game short exercises performed by players at a maximal and high intensity (sprinting, striding) dominated by anaerobic energy processes are intertwined with activities of moderate and low intensity (walking, jogging) characterized by aerobic energy processes [18,19]. However, to the best of our knowledge, no studies analysed the players’ activities during the match to estimate the probability of success of a soccer team. Moreover, factors as shots, shots on target, goal attempts, assists, and turnovers have not previously been investigated as factors influencing positions of the final ranking.

Authors that have analyzed the total distance covered and speed among players presented divergent results. In particular, teams such as the Brazilian League, English Premier League, Australian League, European Champions League, and Spanish League had no differences in the high intensity activity between first and second halves; contrarily, in English, Italian and Danish Leagues, high intensity activity decreased in the second half [10,12,13,20,21]. Barbero-Alvarez et al. [22] analysed Spanish players during the first half and second half of the match and found that players covered a mean value of 118.4 meters/min (first half) and 110.5 meters/min (second half), respectively. First-half events such as substitutions, tactical changes, sprint activities, and total shots could explain this data. The intensity of team sports was classified by other authors in distance covered in different velocity ranges [22,23]: Players covered distance principally at low velocities and high intensity running decreased in the second half of the match. However, a significant increase in the high-intensity running percentage was found during the out-of play periods from the first half to the second half: In the second half players try to perform a greater number of set attacking piece plays from corner kicks, kick-ins, or free kicks [24,25,26].

In the present study, no significant differences among teams in the total distance covered and speed were observed, while statistically significant differences for the percentage of jogging, running and sprint activities were reported. The examination of distances and speeds alone can misrepresent the real activity during a match, living important factors such as playing position, effect on the intensity of play, medium accelerations, medium decelerations, and high decelerations and situational factors. The covered distance may be related to the player’s position on the pitch. The longest mean distance in a match is shown to be covered by midfielders, followed by defenders and forwards. The quality of the distance covered seems also important because more distance at high-intensity running and sprinting is performed by elite players compared with other players [27].

Parameters that were most significantly associated with the probability of being in the first positions of the final ranking were investigated by dividing the population into teams that obtained the first three positions of the final ranking, teams that obtained a final ranking from the 4th to the 6th position, teams that ranked from the 7th to the 18th position, and teams that relegated from the “Serie A” championship. Teams that obtained the first three positions of the final ranking made a high percentage of sprint and jogging activities, and a lower percentage of running activity. Moreover, in the same population, total shots, shots on target, goal attempts, assists, and turnovers were higher compared to the other subgroups. The presence of significant differences in total shots, shots on target, goal attempts, assists, and turnover among teams is one of the most robust findings in this analysis: This data could be analyzed to identify a winning profile.

According to previous findings [11,28], we observed that lower-ranked Italian “Serie A” teams covered significantly greater distances in the running activity. This phenomenon may be a direct consequence of the player’s attempts to regain the ball, and perhaps their inability to maintain ball possession. Running activity was also found to be negatively associated with shots, goal attempts and assists, as demonstrated by the correlation analyses between physical performance and parameters related to the match statistics. On the contrary, the percentage of the sprint activity resulted positively associated with shots, goal attempts, assists and steals, suggesting that the explosive start of the run, the ability to increase speed over the covered distance and reaching peak speed are among the key components affecting the players’ effectiveness during a match.

The results of the present study demonstrate that the best predictor for the team’s success at the end of the season is the higher ratio of total shots, shots on target, goal attempts, assists, and turnover. Indeed, our results suggest that the sprint activity as well as total shots, shots on target, goal attempts, and assists were associated with a higher probability of reaching the first three positions and >9 goal attempts per match determined a seven-fold increased possibility of reaching the first three positions. On the other hand, the probability of being in the first six positions of the final ranking seemed to be less influenced by running and sprint activities, and more influenced by the match statistics, in particular total shots, shots on target, goal attempts, assists, and steals. The higher turnover of the teams that obtained the first three positions could be explained by higher risks of these teams.

Some limitations are present. First, we did not have access or report data such as agility, vertical jump height distance or sprint times that may vary between athletes. These factors may contribute to the success among athletes, which would ultimately correlate to the team success. The experience level between soccer players was not examined and may have played a factor in our analysis as well. Second, the study population, despite the larger available in the literature, is limited. Larger populations of athletes should be evaluated for the optimal interpretation of the results.

## 5. Conclusions

In conclusion, the present findings indicate that overall technical and tactical effectiveness may have a greater impact than the running activity on the results obtained by the team. In our analysis, the sprint activity, goal attempts, total shots, shots on target and assists were significantly related to an increased probability to achieve the first positions of the final ranking in the Italian football league “Serie A”, being the best predictors for the teams’ success.

## Figures and Tables

**Table 1:** Athletic performances according to the final ranking (One-way univariate ANOVA analysis).

Rank	Total Distance, km	Distance/min, m/min	Jogging, %	Running, %	Sprint, %	Speed, km/h
1st	107.7 ± 4.0	119.6 ± 4.5	27.0 ± 1.7	65.1 ± 1.6	7.8 ± 0.9	6.6 ± 0.2
2nd	109.9 ± 4.5	122.2 ± 5.0	24.4 ± 1.5	66.6 ± 1.4	9.0 ± 0.8	6.9 ± 0.2
3rd	105.4 ± 3.4	117.1 ± 3.8	27.0 ± 1.3	65.1 ± 1.3	7.8 ± 0.6	6.7 ± 0.2
4th	106.6 ± 3.7	118.4 ± 4.1	26.3 ± 1.6	65.5 ± 1.5	8.2 ± 0.9	6.8 ± 0.2
5th	108.0 ± 4.7	120.0 ± 5.3	24.7 ± 1.5	67.3 ± 1.3	8.0 ± 0.7	6.9 ± 0.2
6th	105.2 ± 4.8	116.9 ± 5.4	26.8 ± 2.1	66.0 ± 1.9	7.3 ± 0.9	6.5 ± 0.3
7th	109.2 ± 3.4	121.3 ± 3.7	25.6 ± 2.1	66.0 ± 1.7	8.5 ± 0.9	6.8 ± 0.2
8th	108.0 ± 4.2	120.0 ± 4.7	25.4 ± 1.6	66.8 ± 1.4	7.9 ± 0.8	6.9 ± 0.2
9th	107.3 ± 5.2	119.3 ± 5.8	26.8 ± 2.1	64.8 ± 3.4	8.0 ± 0.7	6.7 ± 0.3
10th	107.6 ± 3.6	119.5 ± 4.0	25.6 ± 1.7	66.0 ± 1.8	8.4 ± 0.8	6.7 ± 0.2
11th	104.6 ± 5.7	116.3 ± 6.4	27.0 ± 2.0	65.0 ± 1.8	8.0 ± 0.9	6.5 ± 0.3
12th	107.7 ± 4.8	119.6 ± 5.3	25.9 ± 1.8	66.2 ± 1.8	7.9 ± 0.7	6.8 ± 0.2
13th	107.0 ± 5.0	118.9 ± 5.6	26.8 ± 1.6	65.4 ± 1.5	7.8 ± 0.7	6.7 ± 0.2
14th	108.6 ± 3.3	120.6 ± 3.7	25.3 ± 1.4	67.0 ± 1.4	7.6 ± 0.6	6.8 ± 0.2
15th	107.5 ± 5.1	119.5 ± 5.7	26.3 ± 1.8	65.6 ± 1.8	8.1 ± 0.7	6.8 ± 0.2
16th	105.1 ± 4.2	116.8 ± 4.7	26.8 ± 1.6	65.0 ± 1.5	8.3 ± 0.8	6.6 ± 0.2
17th	107.8 ± 4.0	119.7 ± 4.4	25.2 ± 1.6	67.2 ± 1.6	7.6 ± 0.8	6.8 ± 0.2
18th	107.4 ± 4.5	119.3 ± 5.0	24.8 ± 1.6	66.3 ± 3.0	8.4 ± 0.9	6.7 ± 0.2
19th	106.9 ± 3.5	118.8 ± 3.9	25.7 ± 1.7	66.6 ± 1.5	7.7 ± 0.9	6.8 ± 0.2
20th	107.8 ± 4.7	119.8 ± 5.25	24.6 ± 1.7	77.7 ± 1.8	7.7 ± 0.9	6.9 ± 0.2
*p*-value	0.322	0.322	<0.0001	<0.0001	<0.0001	0.238

**Table 2:** Match statistics according to the final ranking (One-way univariate ANOVA analysis).

Rank	Total Shots, *n*	Shots on Target, *n*	Goal Attempts, *n*	Assists, *n*	Turnovers, *n*	Steals, *n*
1st	12.7 ± 4.6	7.0 ± 3.3	10.6 ± 4.1	4.4 ± 2.3	35.5 ± 8.2	25.3 ± 6.1
2nd	14.6 ± 4.5	8.1 ± 3.1	10.9 ± 4.0	3.8 ± 2.1	38.1 ± 12.0	26.5 ± 8.5
3rd	14.4 ± 4.4	7.5 ± 2.3	11.6 ± 4.1	4.8 ± 2.4	33.9 ± 9.7	25.2 ± 7.9
4th	10.4 ± 3.7	6.1 ± 2.8	8.1 ± 3.4	3.2 ± 2.4	34.0 ± 9.6	27.9 ± 9.1
5th	11.3 ± 4.5	6.5 ± 3.0	8.7 ± 4.6	3.1 ± 1.9	32.2 ± 10.1	27.8 ± 9.0
6th	11.7 ± 4.7	6.6 ± 2.9	8.7 ± 3.5	2.8 ± 1.9	32.7 ± 7.5	25.9 ± 8.0
7th	13.5 ± 4.0	6.7 ± 2.7	10.4 ± 4.1	3.4 ± 1.8	35.3 ± 10.9	26.5 ± 7.6
8th	12.9 ± 4.9	7.0 ± 2.8	8.6 ± 3.9	2.8 ± 2.0	36.0 ± 11.6	22.9 ± 6.9
9th	11.4 ± 4.6	5.6 ± 2.9	7.9 ± 4.5	2.9 ± 2.3	31.2 ± 9.0	24.5 ± 7.6
10th	9.0 ± 3.4	5.2 ± 2.3	6.5 ± 2.9	2.3 ± 1.4	33.2 ± 8.8	23.2 ± 7.4
11th	10.4 ± 3.6	5.3 ± 1.9	7.1 ± 3.1	2.8 ± 2.0	33.9 ± 9.7	24.4 ± 8.6
12th	8.6 ± 4.3	5.2 ± 3.3	6.5 ± 4.4	2.7 ± 1.9	34.8 ± 12.4	25.4 ± 9.1
13th	8.6 ± 3.0	4.6 ± 2.1	5.9 ± 3.0	2.2 ± 1.8	30.7 ± 9.3	26.1 ± 9.3
14th	8.2 ± 3.5	4.0 ± 2.1	5.8 ± 3.0	2.3 ± 1.8	31.7 ± 8.3	24.7 ± 8.6
15th	8.6 ± 3.8	4.7 ± 2.6	6.3 ± 3.8	1.8 ± 1.7	29.8 ± 9.5	22.7 ± 5.5
16th	9.3 ± 3.6	4.5 ± 1.7	6.8 ± 3.0	2.6 ± 1.8	33.7 ± 9.5	25.9 ± 8.1
17th	8.0 ± 3.8	4.3 ± 2.3	4.9 ± 2.9	1.9 ± 1.6	27.7 ± 8.8	23.6 ± 6.3
18th	7.6 ± 3.3	3.8 ± 2.3	5.3 ± 3.1	1.8 ± 1.6	33.5 ± 9.7	23.5 ± 8.7
19th	7.3 ± 3.2	3.7 ± 2.2	4.6 ± 2.0	1.4 ± 1.4	29.6 ± 8.8	21.4 ± 4.8
20th	8.9 ± 3.4	4.8 ± 2.1	6.3 ± 3.1	2.0 ± 1.6	36.2 ± 12.2	24.7 ± 7.9
*p*-value	<0.0001	<0.0001	<0.0001	<0.0001	<0.0001	0.022

**Table 3:** Athletic and match performances according to the different positions of the final ranking (Kruskal-Wallis test for comparisons among groups).

Variable	1st–3rd Position	4th–6th Position	7th–17th Position	18th–20th Position	*p* for Trend
Jogging, %	26.1 ± 1.9	25.9 ± 1.9	26.1 ± 1.8	25.0 ± 1.7	<0.0001
Running, %	65.6 ± 1.6	66.2 ± 1.7	65.9 ± 2.0	66.9 ± 2.3	<0.0001
Sprint, %	8.2 ± 1.0	7.8 ± 0.9	8.0 ± 0.8	7.9 ± 0.9	0.025
Total Shots, *n*	13.9 ± 4.6	11.1 ± 4.3	9.9 ± 4.2	7.9 ± 3.4	<0.001
Shots on Target, *n*	7.5 ± 2.9	6.4 ± 2.9	5.2 ± 2.6	4.1 ± 2.2	<0.001
Goal Attempts, *n*	11.0 ± 4.1	8.5 ± 3.8	7.0 ± 3.8	5.4 ± 2.8	<0.001
Assists, *n*	4.3 ± 2.3	3.0 ± 2.1	2.5 ± 1.9	1.7 ± 1.5	<0.001
Turnovers, *n*	33.8 ± 10.1	33.0 ± 9.1	32.5 ± 10.1	33.1 ± 10.6	0.022
Steals, *n*	25.7 ± 7.5	27.2 ± 8.7	24.5 ± 7.8	23.2 ± 7.4	<0.001

**Table 4:** Correlation analyses between physical performance and parameters related to the match statistics. Data are reported as R (*p*-value).

Variables	Speed	Jogging	Running	Sprint
Total Shots	−0.01 (0.819)	0.11 (0.004)	−0.16 (<0.001)	0.13 (<0.001)
Shots on Target	0.03 (0.418)	0.06 (0.129)	−0.09 (0.012)	0.10 (0.005)
Goal Attempts	−0.03 (0.475)	0.12 (0.002)	−0.19 (<0.001)	0.15 (<0.001)
Assists	0.04 (0.319)	0.08 (0.023)	−0.13 (<0.001)	0.10 (0.007)
Turnovers	0.09 (0.015)	−0.10 (0.005)	0.07 (0.068)	0.08 (0.029)
Steals	0.14 (<0.001)	−0.10 (0.005)	0.07 (0.042)	0.06 (0.083)

**Table 5:** Univariate logistic regression analysis for achieving the first three and six positions of the final ranking according to the 3rd tertile of the distribution of athletic and match performances.

Variables	First 3 Positions			First 6 Positions		
	OR	95% CI	*p*-Value	OR	95% CI	*p*-Value
Jogging > 26%	1.16	0.78–1.73	0.464	1.20	0.88–1.64	0.254
Running > 66%	0.57	0.38–0.86	0.007	0.74	0.54–1.01	0.061
Sprint > 8%	1.77	1.18–2.65	0.006	1.14	0.83–1.59	0.421
Total Shots > 12	4.55	3.01–6.89	<0.001	3.34	2.39–4.66	<0.0001
Shots on Target > 6	3.60	2.39–5.43	<0.001	3.33	2.40–4.60	<0.0001
Goal Attempts > 9	7.21	4.69–11.09	<0.001	4.49	3.19–6.30	<0.0001
Assists > 3	4.45	2.94–6.74	<0.001	2.81	2.02–3.90	<0.0001
Turnovers > 37	1.48	0.97–2.23	0.066	1.15	0.82–1.60	0.419
Steals > 27	1.44	0.95–2.19	0.085	1.81	1.30–2.52	<0.0001
